# CHANS-Law: preventing the next pandemic through the integration of social and environmental law

**DOI:** 10.1007/s10784-022-09566-7

**Published:** 2022-03-13

**Authors:** Kirsten Davies, Michelle Lim, Tianbao Qin, Philip Riordan

**Affiliations:** 1 University of New England Law School, Armidale, Australia; 2 Macquarie Law School, Macquarie University, Sydney, Australia; 3 Research Institute of Environmental Law (RIEL), Wuhan, China; 4 School of Law, Wuhan University, Wuhan, China; 5 China Society of Environmental and Resources Law, Wuhan, China; 6 Wildlife Without Borders, London, UK; 7 Department of Biological Sciences, University of Southampton, Southampton, UK; 8 Marwell Wildlife, Winchester, UK

**Keywords:** International environmental agreements, Epidemics and pandemics, One-Health, COVID-19

## Abstract

Zoonotic viruses have sacrificed hundreds of millions of people throughout human history. There are currently 1.7 million unidentified viruses estimated to be circulating in mammal and bird populations. It is foreseeable that in the near future, another of these will transmit to people, heralding the start of the next pandemic—one potentially more deadly than COVID-19. At the core of this article is a call for pre-emptive protection of the natural environment and its regenerative systems as the first fundamental step in the prevention of future epidemics and pandemics. While zoonoses originate in nature, the predominant legal discipline, managing these crises, is international health law which is invoked reactively once an outbreak has been reported. In this paper, we identify the need for a legal shift in epidemic and pandemic responses. In particular, we call for the incorporation of international environmental agreements to prevent the initial viral spillover from animal to human populations. We propose a strategy of strengthening existing agreements and a coupling of legal disciplines, such as health and environmental law, emphasizing the need for synergies across legal disciplines to enhance the emergence and management of future pandemics and epidemics. We introduce Coupled Human and Natural Systems (CHANS) Law to frame the required integration across legal instruments to regulate inextricably human-nature connections and advocate for the development of a *Convention on Epidemics and Pandemics.*

## Introduction

Zoonotic diseases, pathogens which enter human populations via animal hosts, are considered one of humanity’s greatest challenges (Johnson et al., 2020). Approximately 60% of infectious diseases in humans are zoonotic, and 75%of emerging infectious diseases (EIDs) are of animal origin (Robbins, 2012; Taylor et al., 2001).

In epidemiology, an ‘epidemic’, refers to the progress of a pathogen spreading within a community of susceptible (i.e., previously uninfected) individuals (ReliefWeb, 2008). In a ‘pandemic’, the disease will have spread worldwide, not just isolated to a geographical community, and be simultaneously present in both hemispheres (Kelly, 2011).There are numerous examples of zoonotic epidemics and pandemics (see Table 1). Human Immunodeficiency Virus/Acquired Immunodeficiency Syndrome (‘HIV/AIDS’), for example, was initially transmitted into the human population via poachers killing infected chimpanzees. HIV/AIDS, has subsequently resulted in the deaths of approximately 32 million people since it was first identified in 1981 (UNAIDS, 2020). A recent example of a zoonotic pandemic is the novel coronavirus (‘SARS-CoV-2’), which, better known as COVID-19, spread globally through nations and communities from late 2019 onwards (WHO, 2021c). At the time of writing this paper, Omicron a variant of COVID-19 is infecting populations across the planet.

**Table 1:** Examples of human-nature interactions and the transmission and impacts of zoonotic disease

Disease	Year	Human-nature interaction	Human impact@@
H1N1 (‘Spanish flu’, Swine flu)	1918, 2009	Thought to have emerged first in wild birds then circulated within human and domesticated pig populations (Hoag, 2014)	‘Spanish’ Flu: estimated 17 million human deaths (Spreeuwenberg et al., 2018) Swine flu: estimated 105,000- 395,000 human deaths (WHO, 2020a)
HIV/AIDS	1920’s (spillover thought to have occurred); 1980’s start of global spread	First identified in the Democratic Republic of Congo, Africa, in the 1920’s after poachers sacrificed chimpanzees, that were deemed to be living too close to human settlements (Sharp & Hahn, 2010)	Estimated 32 million human deaths to date (UNAIDS, 2020)
Zika	Identified in Uganda 1947 Latin American outbreak 2015	Transmission from *Aedes* spp. Mosquitoes (WHO, 2020a)	Vertical transmission can lead to severe birth defects, such as congenital malformations, and microcephaly (abnormal brain development) (Ladhani, O'Connor, Kirkbride, Brooks, & Morgan, 2016)
Ebola	Discovered 1976; 2014–2016 West African Outbreak	The virus probably originated in wild insectivorous bats, with the first transmission event traced to a two-year old boy in Guinea (Saéz et al., 2015). Transmissions may also have occurred through contact with the animals’ bodily fluids, during slaughter, and the contamination of crops, through their waste droppings (WHO, 2020c; Reichler et al., 2020) Developed and spread in remote villages close to tropical rainforests, which were the natural habitat of these animals (WHO, 2020c)	Fatality rate ~ 50%, could be as high as 88% (Saéz et al., 2015) WHO estimate 15,267 deaths since 1976 (WHO, 2021c). In 2006 Riordan et al. noted substantial under-reporting (Riordan et al., 2006)
Avian influenza (H5N1)	1997 (identified in poultry) 2003 (widespread transmission)	Thought to originate in wild birds, entered human populations via intensive poultry farming (Jones et al., 2013)	Economic costs of deaths of millions of poultry birds 455 confirmed human deaths (WHO, 2020b)
SARS-CoV-1	2002–2004	Believed to have originated from a colony of bats in China (Xu et al., 2004). The virus ‘jumped’ the species barrier and was transmitted to humans via contact with the animal’s bodily fluids or contaminated fruits the bats may have been in contact with. The virus mutated sufficiently to be transmitted from human to human (Li et al., 2006)	Fatality rate ~ 9.6%; 774 confirmed deaths (Li et al., 2006)
Middle East respiratory syndrome (MERS-CoV)	2012	Virus originated in bats and transmitted to dromedary camels through unknown means. Entered human population as the result of contact with infected camels (Killerby et al., 2020)	Identified in 27 countries. Fatality rate ~ 35%. 2,494 confirmed cases and 858 deaths (WHO, 2020d)
COVID-19	2019- ongoing	The 2021 WHO Report found, in response to an expert investigation surrounding the original source of the virus in China, that: ‘direct zoonotic spillover is considered to be a possible-to-likely pathway; introduction through an intermediate host is considered to be a likely to very likely pathway.’ (p. 9 WHO, 2021b)	Mortality rate ~ 4% (Dong et al., 2020) As of January 2022, estimated 5,518,343 deaths, 222 countries with cases (WHO, 2022)

The incidence of zoonotic epidemics and pandemics is predicted to increase (Carroll et al., 2018). This is largely attributable to global human population growth and changes in land-use, agricultural expansion, urbanization and intensive animal farming (Keusch et al., 2009; IPBES, 2020). Further, the impacts of climate change are expected to exacerbate the conditions leading to future pandemics (Curseu et al., 2010). While epidemics and pandemics do not discriminate between social groups, the outcomes often place vulnerable people at greatest risk. For example, higher disease risk in the tropics, when combined with higher rates of poverty, presents challenges at the interface between the goals of human wellbeing and the conservation of nature (Riordan et al., 2006). Thus, we see an interlinking nexus between biophysical systems, including climate and biodiversity, and human social and economic systems. Thus, an integrated legal response is needed which appreciates the embeddedness of human systems within nature (Govind, 2020).

In this article, we discuss the inseparable relationship between people and nature in terms of coupled human and natural systems (‘CHANS’). CHANS are defined as ‘integrated systems in which people interact with natural components’ (Liu et al., 2007). The indivisibility of social and ecological systems (Folke et al., 2005), is reflected in the environmental drivers of epidemics and pandemics and their resultant human impacts.

Minimizing the emergence of future epidemics and pandemics requires a strategy which couples legal disciplines, which we present here as a CHANS approach to law (‘CHANS-Law’). That is, an approach to law that appreciates, and actively coordinates, the intrinsically interconnected nature of social and ecological issues and by extension, the instruments and institutions which govern these issues. Recognizing that zoonotic diseases originate in nature, we investigate the current and future role of international environmental agreements in minimizing, or preventing, their emergence and subsequent transmission to people (IPBES, 2020).

The central question investigated in this paper is: **‘**Can a CHANS-Law approach assist in the prevention and management of future epidemics and pandemics emerging from zoonotic sources?’ To respond to this question, we first expand on the CHANS approach and present the case as to how it can inform the law. We then use the global prevention and management of epidemics and pandemics as a case-study to examine how a CHANS-Law approach could be operationalized. We progress to examine international health and environmental agreements in the context of their existing and potential contributions to preventing zoonotic disease. The paper concludes by proposing changes to current legal frameworks to embrace proactive, along with reactive responses. This includes advocating for a greater role for international environmental law and the need to develop a *Convention on Epidemics and Pandemics.*

## Coupled Human and Natural Systems (CHANS), zoonotic disease and CHANS-Law

The overarching conceptual framework that we bring to the international regulation of zoonotic disease and epidemic and pandemic preparedness and prevention is the Coupled Human and Natural Systems (CHANS) approach that emphasizes the inextricable links between human and natural systems. In this section, we expand on the characteristics of a CHANS approach and discuss its relevance in the context of epidemics and pandemics of zoonotic origin. The section concludes by setting out the CHANS-Law approach and briefly describing the comparative doctrinal approach we adopt throughout the rest of the paper.

### Coupled Human and Natural Systems (CHANS)

(CHANS) are integrated systems where humans and nature interact (Liu et al., 2007).Systems are ‘coupled’ when the interconnected flows of information, material or energy result in effects in one system that cannot be meaningfully explained without understanding the corresponding system (Alberti et al., 2011). Thus, feedbacks within coupled systems can dramatically amplify small changes across the system (Capra & Luisi, 2014). Tourism provides an illustrative example of the complex feedback loops within CHANS (Liu et al., 2007). Healthy ecosystems provide the foundation of tourism in many areas and, therefore, also contribute significantly to the economy. However, economic development to support tourism can often degrade the ecosystems which tourism relies on. This in turn can have detrimental economic impact (Liu et al., 2007).

A CHANS approach, in turn, seeks to integrate social and natural science disciplines to understand these complex human-nature interactions (Davies, 2019). Such approaches are more important in the rapidly unfolding ‘Anthropocene’, an era characterized by 'human dominated ecosystems' (Vitousek et al., 1997; Gallagher & Carpenter, 1997). Meanwhile, rapid changes across biophysical systems have powerful impacts on human systems.

However, such impacts are felt very differently with those in extreme poverty the most vulnerable to biophysical threats and global changes in resource availability (Kotchen & Young, 2007). This underscores the intertwined environmental, social, economic and equity issues of interconnected human-nature systems.

### CHANS and the emergence of zoonotic disease

Escalating pandemic risk results from the increased probability of human-animal interactions (Johnson et al., 2020) (see Table 1). Zoonotic disease spread can arise from unsustainable environmental exploitation which disrupt otherwise stable natural interactions between wildlife and microorganisms, including bacteria and viruses (e.g., Riordan et al., 2011). Along with the exploitation of the natural environment, increased contact between humans, livestock and wildlife, is growing and with it, the risk of disease spillover into human populations (IPBES, 2020). This manifests in the form of deforestation and agricultural intensification (Jones et al., 2013); increased intensive livestock production as well as the exploitation of, and trade in, wildlife (Marco et al., 2020; Karesh et al., 2005). These unsustainable human-nature interactions heighten the risk that diseases in animals will increasingly transmit into human populations (Jones et al., 2013). For example, reporting of infectious viruses transmitting from bats to humans is set against a long history of such transmission, as in the case of rabies. More current examples include Marburg virus in Egypt, and Hendra virus in Australia. By virtue of their ecology and life-histories, bats maintain a relatively high diversity of viruses, including zoonotic species (Luis et al., 2013). The growth of human settlements and increased deforestation of natural bat habitats have resulted in people and bats living in closer proximity, thereby increasing the risk of virus transmission (Kelly, 2011). Further examples of human- nature interactions and the emergence of zoonotic disease are presented in Table 1.

Habitat destruction and encroachment by people create new entry points for the spread of disease into human populations (IPBES, 2020). Changes to ecosystems and decreased biodiversity can enhance the risk of animal diseases entering human populations (IPBES, 2020; Jones et al., 2013; Ostfeld, 2009). Reduced levels of biodiversity bring potential wildlife hosts, such as animals and birds, into closer proximity with insect vectors such as ticks and mosquitos—thus increasing the spill-over risk (Ostfeld, 2009). Intensive farming further increases the risk of zoonotic disease emergence, by housing large numbers of domesticated species, often with suppressed immunity, in close quarters, creating conditions for microparasites to rapidly spread and evolve greater virulence (Jones et al., 2013). The close proximity of domesticated animals with humans creates ample opportunity for novel diseases to enter human communities. Enhanced global trade, as well as illegal movement of produce, including from wild sources, further increases the potential for zoonoses to reach new territories.

The illegal global trade of wildlife is worth billions of dollars annually, and puts many species of both flora and fauna at risk (). Wildlife trade inevitably increases the risk of zoonotic disease spread, by increasing the number of potentially infectious contacts. At the same time, the increased demand for meat consumption and the globalization of the food trade also enhances the risk of pandemics (IPBES, 2020). Through protecting ecosystems and wildlife habitats, the likelihood of close contact between wildlife and human settlements is reduced, thereby limiting potential zoonotic infectious risks and the chances of the spread of zoonotic diseases (UNEP, 2020).

There are also complex feedback mechanisms across and between biophysical systems with emergent synergies between the effects of land-use conversion, climate change, biodiversity loss and emerging diseases (IPBES, 2020), both infectious and non-infectious. For example, deforestation leads to an increase in the effects of climate change and the loss of biodiversity. Climate change causes decreases in arable land availability and biodiversity. This in turn, leads to an increase in the short-term exploitation of resources leading to increased deforestation (Shearer, 1994). There has been a noted connection between climate change and the spread of zoonotic diseases, where increased outbreaks are likely as the planet warms (Curseu et al., 2010). Climate change will have direct impacts on the distributions of zoonotic disease, including through an increased range of suitable habitat for insect vectors such as mosquitoes and ticks leading, for example, to the northward spread of malaria in Europe (Lafferty, 2009). Singh et al. (2021) concluded (p. 12) ‘that weather variables, as well as forest and biodiversity conservation, have the potential to influence human, zoonotic and emerging pathogen diversity in the near future.’

The reciprocal relationships within CHANS can be captured in Eco-Health and One-Health initiatives, for example, which recognize the impacts on health outcomes, and that animal and human health need to be understood in their ecological context (Bunch, 2016; Harrison et al., 2019). This is particularly evident in a pandemic context, recognizing that human-nature relationships underpin disease emergence and that the rapid increase in zoonotic disease outbreaks is directly linked to increased human-animal interactions, thereby also presenting potential solutions (IPBES, 2020).

### A CHANS-Law approach

There is a wealth of the literature (Stephens, 2006; Scott, 2011; Kotze, 2014) highlighting the fragmented nature of international law across multiple international instruments. There remains, however, the need for scholarship which sets out an integrated legal framework which reflects the interconnected nature of human-nature systems. To our knowledge, despite the widespread engagement with the CHANS concept in understanding social-ecological systems (the seminal paper by Liu et al., 2007) for example has close to 1000 citations, no previous work recommends the CHANS approach as a conceptual lens for identifying synergies across international law. Thus, there is a critical need to grow the legal dimension of a CHANS approach (Folke et al., 2005) in the form of CHANS-Law. We define CHANS-Law as a method of engaging multiple legal disciplines to address coupled human-nature challenges. The introduction of a CHANS framework across international agreements would see various legal fields work synergistically, with far reaching global application in areas ranging from climate change, to freshwater management and to broad applications in the fields of human health. In the context of epidemics/pandemics, a CHANS-LAW approach emphasizes the particular need for environmental and health law to act together.

In the sections that follow, we highlight the reactive and siloed nature of existing legal responses to epidemics and pandemics. We also emphasize the role those international environmental agreements can play, within a CHANS-Law framework, to bring an international law approach which not only reacts to the emergence of disease but importantly, facilitates coordination across health and environmental law regimes to address the root causes of disease spillover. To achieve this, we adopt a doctrinal approach.

Doctrinal legal research is the key means by which legal scholars and practitioners engage with legal instruments. At is core, doctrinal legal scholarship concerns determination of what the law is (i.e., not what it should be). The key objective of such an approach is the interpretation of the legal text in question. Here, legal instruments such as treaties, legislation and case-law are examined to determine their meaning and the extent to which the law requires action or inaction in particular contexts (van Hoecke, 2011). Doctrinal legal scholarship, therefore, consists of the systematic examination of legal rules that stem from legal documents and principles as well as the relationships between these rules (Pearce et al., 1987).

In the sections that follow, we examine the scope of relevant international health, environmental and biosecurity agreements in conjunction with key international environmental law principles. Having examined the interrelationships within existing law we shift to a more reformist methodology which sets out how we recommend the law *should* develop to facilitate the integrated CHANS-Law approach called for above.

## International legal responses to pandemics and epidemics

International legal and management responses to epidemics and pandemics are largely reactive, coming in to play once an outbreak has been identified. Responses primarily sit within the human health domain (Mcinnes, 2015). The World Health Organization (‘WHO’) manages the monitoring of potential outbreaks, containment of disease, and building the core health capacity and infrastructure of individual countries (Pang, 2016). There are few binding international agreements pertaining to epidemics and pandemics. Environmental concerns relating to their prevention are particularly neglected. While the *WHO Constitution* asks Member States to provide to the World Health Assembly, epidemiological reports and statistics pertaining to health (United Nations, 1948), in essence, there is only one legally binding instrument, the *International Health Regulations* (2005) (‘IHRs’). The IHRs instrument was developed in 1969 by the WHO in response to yellow fever, cholera, and plague pandemics. The IHRs were later revised, due to the SARS epidemic in 2003 (Simpson & Thompson, 2005), and were in force by 2005 (WHO, 2005). The IHRs are binding on all Member States to the WHO and were established to:

prevent, protect against, control and provide a public health response to the international spread of disease in ways that are commensurate with and restricted to public health risks, and which avoid unnecessary interference with international traffic and trade (WHO, 2005).

There are three methods through which countries must alert the WHO of potential outbreaks. The first is notification. The IHRs set out in Article 6 how countries are required to notify the WHO of a potential epidemic or pandemic within 24 h of an outbreak (WHO, 2005). The second method is ‘consultation’ (WHO, 2014). This is used when there is insufficient evidence within a country to determine whether there is a potential outbreak. The country, through this method, must initiate confidential consultations with the WHO to seek advice on whether domestic measures should be taken (WHO, 2014). The final method is ‘reporting’ (WHO, 2014). This is done through the National IHR Focal Point, within 24 h of an identified outbreak. This method is used when a country receives evidence of a potential outbreak in another country, which may pose an international risk. The reporting country would need evidence through imported goods or persons of the spread. The WHO is able, at any time, to request countries confirm or deny unofficial reports of potential outbreaks in their country (WHO, 2014).

The IHRs are reactive, mainly focusing in the past on cholera, plague and yellow fever. Many emerging diseases, such as the Hendra virus, are not directly addressed. The IHRs provide little, if any, incentive for countries to self-report. Rather than establishing its own preventative measures, the WHO only responds once reporting has occurred. Some further limitations posed by international health law include the ‘free-ride’ principle (Giesecke, 2003), where developed countries receive information that there is a potential outbreak in a developing nation, but may be less likely in the reverse situation, to notify the WHO of a potential outbreak (Giesecke, 2003). Secondly is the so-called ‘prisoner’s dilemma’ problem, where countries may act in their own self-interest and at the expense of the other. For example, should a country notify the WHO that there is a potential pandemic, that country will likely suffer reduced trade and tourism (Giesecke, 2003). Linked to this is the concept of ‘temporal asymmetry’, where a country will quickly reduce trade and tourism to protect itself, then slowly increase economic activities once the threat has passed (Giesecke, 2003).

A common trend in the management of disease outbreaks is that responses are commonly reactive with a focus, almost exclusively, on health-related laws and institutions (Loh et al., 2015).Despite the recognized connection between the environment and the spread of HIV/AIDS (Talman et al., 2013), environmental factors have largely been ignored in the regulatory and governance space. Similarly, the SARS epidemic was managed internationally by the IHRs, the WHO and the operational arm of the WHO, the Global Outbreak Alert and Response Network (‘GOARN’), working in tandem with domestic health regulatory bodies (Hung, 2003; World Health Assembly, 2003). In the case of bovine tuberculosis (*Mycobacterium bovis*), insufficient awareness of environmental context and wildlife host species behavior and ecology in management responses have led to control measures actually increasing the spread of disease (Riordan et al., 2011).

Additionally, the main strategy for managing *H5N1* internationally has been the WHO, working in tandem with domestic health authorities, the Food and Agriculture Organization (‘FAO’), and the World Organization for Animal Health (‘OIE’) (WHO, 2019). The H1N1 pandemic was also managed internationally through the utilization of the IHRs and the GOARN system (WHO, 2019).

In the management of MERS-CoV, the WHO worked alongside public and animal health specialists, domestic health bodies, the FAO, and the OIE. The WHO also utilized the IHRs to determine appropriate measures to be taken internationally (WHO, 2019). Ebola was managed through the WHO’s Emergency Response Framework. The IHRs were considered a ‘last resort’ in managing this virus, however, they were ultimately required (Honigsbaum, 2017). The Zika virus is managed by the WHO and the IHRs to develop and strengthen monitoring systems and assist domestic health care bodies to prevent further transmission (WHO, 2014). COVID-19 is currently being managed through the WHO creating a preparedness and response plan in line with the IHRs and domestic health bodies (WHO, 2021a).

Current pandemic strategies, focused on the development of treatments and vaccines, are largely directed at controlling disease once it appears in human populations (IPBES, 2020). There remains little emphasis on the environmental origins of disease, and the interactions between people and nature that drive their emergence as epidemics and pandemics. Nevertheless, there appears to be positive shifts toward greater acknowledgement of the environmental components of pandemic prevention and response spurred by COVID-19. Examples include the *United Nations Environment Program* ('UNEP') guidelines and biosecurity legislation applied in some domestic contexts such as Australia, New Zealand and Canada. Additionally, in 2020 China released its Decision of the Standing Committee of the National People's Congress on the Total Prohibition of Illegal Wildlife Trade, Elimination of the Consumption of Wild Animals, and the Effective Protection of Human Health (‘Standing Committee Decision on Wildlife Trade and Consumption’) which provides instruction on revision of the *Wildlife Protection Law* (National People’s Congress of the People’s Republic of China, 2020) This trend suggests increasing acknowledgement of the environmental component of the spread of zoonotic disease. However, there remains a scarcity of specific international legal instruments linking environmental protection to the prevention of epidemics and pandemics. The section that follows, therefore, explores the potential role that international environmental law generally, and international environmental agreements in particular, can play as a part of a CHANS-Law framework to facilitate a proactive international approach to epidemics and pandemics which addresses the root causes of pandemic emergence.

## The role of international environmental law and agreements

International environmental law principles have much to offer in enhancing current legal approaches to epidemics and pandemics. The principles of prevention, precaution and participation, are of particular importance. We discuss each in turn below before turning to particular International Environmental Agreements: the Convention on the International Trade in Endangered Species (CITES), the Convention on Biological Diversity (CBD) and the United Nations Framework Convention on Climate Change (UNFCCC). We conclude the section by considering how the international biosecurity framework provides a useful linking mechanism for a CHANS-Law approach to addressing epidemics and pandemics.

### Principles of international environmental law

#### Principles of prevention and precaution

The Prevention Principle is based on the premise that damage should be avoided thus reducing or eliminating risk prior to it becoming a problem (de Sadeleer, 2002). This principle is endorsed by multiple treaties and recognized as a principle of customary international law (de Sadeleer, 2002). The Precautionary Principle, on the other hand, involves ‘the intuitively simple idea that decision makers should act in advance of scientific certainty to protect the environment from incurring harm’ (Raffensperger et al., 1999). The difference between these principles concerns the level of uncertainty relating to the probability of a risk (de Sadeleer, 2002). The Precautionary Principle requires action at an earlier stage, even 'when there is not yet conclusive scientific evidence as to the harmfulness’ (Jaeckel, 2017). It is worth noting that China’s Standing Committee Decision on Wildlife Trade and Consumption recognizes the role of the precautionary principle (National People’s Congress of the People’s Republic of China, 2020). This could potentially provide a model for considering the links between environmental protection and pandemic prevention in an international context (Lan & Qin, 2021).

#### Principle of public participation

The Public Participation Principle states that members of civil society should be able to participate in decision-making processes pertaining to the environment. Public participation has been incorporated in *Rio Declaration* Principles 6–8 with Principle 10 requiring state parties to enable and inspire public participation (Atapattu, 2007). The application of this principle will be critical in future prevention of outbreaks because it will involve communities ‘on the ground’ being educated on the origins and risks of zoonotic disease. Local people are the frontline guardians of nature and will thus play a critical role in preventing the transmission of these pathogens.

### Multilateral environmental agreements

A range of Multilateral Environmental Agreements **(**MEAs) have important roles to play in reducing the likelihood of emerging future epidemics/pandemics. These include biodiversity related conventions (e.g., *Convention on International Trade in Endangered Species of Wild Fauna and Flora* (‘CITES’), the *Convention on Biological Diversity* (‘CBD’) and the *Convention on Migratory Species*). The *United Nations* (‘UN’) *Framework Convention on Climate Change* (‘UNFCCC’), and the *United Nations Convention to Prevent Desertification* are also key to addressing the causes of epidemics/pandemics. With a focus on CITES, CBD and UNFCCC, the role and potential is recognized of other MEAs and the importance of engaging parties to those agreements and Convention Secretariats.

#### Convention on the International Trade in Endangered Species (CITES)

CITES governs the international trade of animals, plants, and products derived from wild flora and fauna that are considered priorities as listed in its three Appendices (United Nations, 1975).In conjunction with CBD, CITES aims to prevent habitat loss while reducing extinction risk (CITES, 2020a, 2020b). A key limitation of CITES in the current context is its focus on endangered species, whereas zoonotic diseases can be carried by both endangered and non-endangered species. Nevertheless, the Convention covers approximately 5800 animal species and has demonstrably reduced the trade in animals listed in its Appendices. This may also have inadvertently reduced the spread of disease (Borsky et al., 2020). Encouragingly, against the backdrop of COVID-19, the CITES Secretariat has pledged to work with parties to examine how the Convention might mitigate the risks of zoonotic disease (Higuero, 2020).

The CITES permit and certificate system, and the establishment of national-level management and scientific authorities, are key to the Convention’s effectiveness (United Nations, 1975). Penalties for not conforming to the requirements, through trade bans and restrictions are a further reason for the Convention’s successful implementation (UNEP, 2006; Goeteyn & Maes, 2011). Illegal trade in CITES protected species can also be reported by the World Customs Organization and INTERPOL (Goeteyn & Maes, 2011). It is one of a handful of treaties which allow third parties (e.g., NGO TRAFFIC Network) to report on non-compliance. Despite its success, fewer than 15% of CITES signatories have legislation that adequately reflect the Convention (Hewitt, 2002). Embedding and enforcing CITES consistently into the domestic laws of states remains a significant challenge.

#### Convention on biological diversity (CBD)

The CBD protects flora, fauna and ecosystems (United Nations, 1993), thereby, indirectly protecting human communities from the transmission of zoonotic diseases. This is because intact natural areas limit the spillover of disease to humans. Over the last 20 years, CBD’s operation has been characterized by a target-based approach (Lim, 2019). The Aichi Targets (2010–2020), under the CBD, consisted of 20 targets aimed at stemming global biodiversity loss and addressing the conservation, sustainable use and equitable sharing of benefits objectives of the Convention. It is likely that in Kunming, China in April 2022 at the second part of the 15th Conference of the Parties to the CBD, CBD contracting parties will agree the Post-2020 Global Biodiversity Framework (‘Global Biodiversity Framework’) the successor targets to Aichi.

There is a lack of explicit acknowledgement in either the Aichi Targets or the first-draft of the proposed Global Biodiversity Framework of the link between the drivers of biodiversity loss and pandemic risk. Nevertheless, targets aimed at addressing contributors to land-use change (Draft Targets 1–4) and wildlife trade (Draft Target 5) are important. However, more is needed to recognize the interconnectedness of social, economic and environmental concerns and to directly address the underlying causes of biodiversity loss such as, unsustainable production and consumption as well as broader issues of trade beyond wildlife trade.

Other opportunities within the Convention pertain to the development of national plans, strategies and programs for the conservation and sustainable use of biodiversity (Article 26) (United Nations, 1993). There is also recognition that such actions need to be integrated through the employment of cross-sectoral approaches into national decision-making (Article 6(b), 10(a) (United Nations, 1993).

#### International climate change regime

The global response to climate change, including through UNFCCC, *Kyoto Protocol* and the *Paris Agreement*—are collectively known as the International Climate Change Regime (‘ICCR’). The UNFCCC was drafted to protect people and nature, as climate change is a direct threat to humanity and nature alike (United Nations, 1992). It was the first international treaty recognizing the need to reduce greenhouse gas (‘GHG’) emissions (United Nations, 1992). The *Paris Agreement* exemplifies CHANS in its objectives to mitigate GHG emissions and foster adaptation measures.^1^ A central concern of the ICCR is the support of developing states and vulnerable communities. The impacts of climate change have been exacerbated by COVID-19 as explained by the Prime Minister of Tonga to the UN General Assembly:

while small island developing states including Tonga contribute to no more than 1% of global greenhouse gas emissions, it is unfortunate that we continue to bear the brunt of this climate injustice. As a result, Pacific Island countries continue to be imperiled by many tropical cyclones of unprecedented magnitude and descriptive nature … and this is while we grapple with the distressing effects of the COVID-19 outbreak (United Nations, 2020).

At the same time, climate change will likely amplify the emergence and spread of vector-borne zoonotic disease (i.e., disease spread to humans via intermediate species such as mosquitoes and ticks) (Naicker, 2011). Meanwhile, Mills et al. (2010) stress the importance of an ecosystem approach to disease prevention which considers ‘the whole environment in which disease occurs’. This underscores the need for a CHANS-Law approach to disease prevention and management in a rapidly changing world.

### Biosecurity

Biosecurity is a key sector at the intersection of environment and health which needs to be integrated into the management and regulation of epidemics and pandemics. It is increasingly recognized that global regulation of plant and animal health, food safety and environmental protection can no longer be regulated in a traditional sectoral-based manner. Nevertheless, international instruments relating to biosecurity continue to lack coherence and therefore do not enable the lens of biosecurity to live up to its potential to facilitate a coordinated and integrated approach across sectors (Outhwaite, 2010).

The aim of biosecurity is to ‘prevent, control and/or manage risks to life and health’ (INFOSAN, 2010). The Food and Agricultural Organization (‘FAO’) recognizes biosecurity as being composed of three sectors: food safety, plant health and life; and animal health and life (FAO, 2001). The World Trade Organization (‘WTO’) *Agreement on the Application of Sanitary and Phytosanitary Measures* (‘SPS Agreement’)is the main international agreement regulating biosecurity (WTO, 1994). The SPS Agreement allows states to implement domestic biosecurity measures to protect human, animal and plant health (World Trade Organization, 1994). However, states are limited to doing so ‘based on scientific principles’ and continued implementation requires ‘sufficient scientific evidence’ (WTO, 1994). States, therefore, rely on standards of the *International Plant Protection Convention* Secretariat as the scientific basis for plant health, the World Animal Health Organization (‘OIE’) in relation to animal health; and for food safety, the Codex Alimentarius Commission (Outhwaite, 2010). In practice, therefore, the SPS Agreement perpetuates traditional sectoral-based siloes. MEA’s on the other hand, do not expressly address issues of biosecurity beyond the important, but limited, context of Invasive Alien Species (Outhwaite, 2010).

Encouraging developments, recognizing the role of biosecurity include the OIE’s promotion of the ‘One-Health’ concept. (World Organization for Animal Health, 2020). However, unless its potential is embraced across the international regulatory sphere, the objectives and potential of biosecurity in the regulation of epidemics/pandemics will continue to be constrained (Outhwaite, 2010).Nevertheless, promising developments which take a more holistic approach have been observed in the domestic context. Countries, such as New Zealand, have revised their legal and regulatory systems to allow for more regular and efficient dialogue between stakeholders, both nationally and internationally (UNEP, 2006). Meanwhile, in response to COVID-19 the Australian Government utilized its powers to manage the pandemic under Sect. 475 of the *Biosecurity Act 2015* (Cth) (Biosecurity Act (Cth), 2015). In 2020, it brought in to legislation, under Sect. 475, *the Biosecurity (Human Biosecurity Emergency) (Human Coronavirus with Pandemic Potential) Declaration 2020* (Australian Government, 2020). Thereby demonstrating the evolution and application of the Act that spans beyond managing plant and animal diseases and pests, by strengthening the human health dimension. This legislation provides an example of the growing need for coupled human and natural law.

## Discussion: advancing a CHANS-Law approach to epidemics and pandemics

In responding to the central question of this paper **‘**Can a CHANS-Law approach assist in the prevention and management of future epidemics and pandemics emerging from zoonotic sources?’ the following developments are proposed.

### Integrate International Environmental Law and Agreements into the Global Epidemic/Pandemic Response

More pandemic/epidemic specific coordination is required within the international environmental governance sphere. This could take the form of dedicated liaison groups across Conventions administered through Secretariats or facilitated through a high-level intergovernmental council or health, environment and trade partnership (IPBES, 2020).

Substantive evidence has been provided in this paper demonstrating that to date international environmental law has played a backseat in the management of zoonotic disease. It has been argued that zoonotic disease emanates from nature and that the disruption of natural cycles is leading to the likelihood of the more frequent emergence of pandemics and epidemics (Curseu et al., 2010; Lafferty, 2009; Singh et al., 2021). Therefore, strengthening existing international environmental law and agreements is essential in terms of minimizing the likelihood of future diseases emerging. Of particular note, is the role of the principles of precaution, prevention and participation underpinning future responses.

Biosecurity, as a framework, may provide a useful means of implementing a CHANS-Law approach in relation to epidemics and pandemics. Such an approach is increasingly important considering the risks which emerge from the exponential growth in global trade and transport and the novel impacts related to new technologies and biophysical shifts such as climate change (Outhwaite, 2010; Sutherst, 2001). Australia, for example, has evoked its Biosecurity Act^2^ to manage COVID 19 and China’s Standing Committee Decision on Wildlife Trade and Consumption recognizes the role of the precautionary principle (National People’s Congress of the People’s Republic of China, 2020).

A key means of preventing the next pandemic, would be to integrate MEAs into the global pandemic response while building collaboration across international environmental instruments. Building on existing MEAs would reduce the time and expense necessary for countries to negotiate and develop a new piece of international environmental law (Palmer, 1992).

### Institutional coordination

There are relatively few environmental actors employed in the international governance of epidemics and pandemics. One of the most notable is the UNEP, which is tasked with addressing environmental concerns, assisting indirectly in the reduction of global pandemics and epidemics, as it aims to increase biodiversity, combat climate change, and more (Andersen, 2020; Peichert, 2007). Additionally, UNEP has created a ‘zoonotic early warning system’ as well as strategies to curb the degradation of ecosystems (Andersen, 2020). Greater explicit linkages are, however, required across Convention Secretariats; national health and environment departments and ministries and with Indigenous and local communities.

### Establish a convention on epidemics and pandemics

The potential of greater coordination across international organizations, Convention Secretariats as well as national and local authorities has been identified. Ideally, epidemic/pandemic responses require a custom-built instrument which implements CHANS-Law and draws together the recommendations set out above. For this purpose, we propose the urgent need for the UN to develop a *Convention on Epidemics and Pandemics* (‘UNCEP’).

This echoes the calls of member states of the WHO have recently highlighted the need for a treaty, such UNCEP when they stated ‘we believe that nations should work together toward a new international treaty for pandemic preparedness and response’(WHO, 2021a).

The current global impact of COVID-19 across all sectors of human life, along with existing epidemics and pandemics, supports the need for such a Convention. Precedents supporting the need for the UNCEP include the preventative *Treaty on the Non-Proliferation of Nuclear Weapons* (United Nations, 1970)*,* and the reactive *Convention on Early Notification of a Nuclear Accident* (United Nations, 1986)*.* This Convention was developed to reduce the likelihood of the mass loss of human lives and to ensure timely reporting when an incident occurred, such as is being experienced through zoonotic disease outbreaks.

The central role of UNCEP could be to explicitly link the drivers of, and responses to, epidemic/pandemic emergence. In other words, such a Convention could facilitate linkages between international health instruments and institutions and those responsible for global environmental protection. The environmental component of such a Convention would focus on: 1/ Reducing the likelihood of potentially zoonotic pathogens (‘PZPs’) transmitting from wildlife reservoirs to people or domestic animals (as seen in the cases of COVID-19, Zika Virus, West African Ebola, MERS-CoV, SARS and HIV/AIDS), and 2/ Reducing the likelihood of PZPs transmitting from domestic animal reservoirs to people, such as H1N1 Swine Flu and Avian Influenza.

The UNCEP would mandate application of the prevention, precautionary and public participation principles discussed above to support rapid responses to reduce the likelihood of the transmission of pathogens. Prevention and participation necessitate recognition that biophysical phenomena such as climate change, land degradation, biodiversity loss and trade interact in ways that amplify their impacts. UNCEP would, therefore, link to other instruments such as CITES, CBD, and UNFCCC in a coordinating and unifying capacity to stem potentially cascading environmental problems directly impacting each other. Further, the integration of existing instruments in a CHANS framework would likely also deliver food security for those in poverty and reduce the need for wild animal consumption. The UNCEP would highlight the role of domestic law and states in both the development, and subsequent enforcement of the Convention.

The critical role of local and Indigenous communities, including their engagement, education and empowerment in preventing an outbreak needs to be acknowledged, together with the importance of Indigenous ontologies forging healthier human-nature relationships. CBD recognizes the need to maintain the ‘knowledge, innovations and practices of Indigenous and local communities’ and the involvement of these communities in the use and application of knowledge, innovations and practices (United Nations, 1993). However, many Indigenous nations consume wild animals due to long-held cultural practices (UN, 2009). The question then arises as to when, and whether, these communities can continue these practices in the context of epidemic/pandemic prevention. This discussion must begin with Indigenous groups worldwide to understand the complexities of this issue in tandem with the maintenance of their beliefs and cultural practices.

The second function of UNCEP would be primarily ‘reactive’ and would sit under the jurisdiction of the WHO and health law, as is presently the case. The importance of the GOARN, working through the IHRs with domestic health law, will be a core function of the UNCEPPP (Hung, 2003; World Health Assembly, 2003). The 2005 revision of the IHRs to protect against ‘emerging diseases’ highlights the growing nexus between preventative and reactive responses (Simpson & Thompson, 2005).

## Conclusion

COVID-19 has claimed over five million lives so far (WHO, 2022) and the economic cost of the pandemic is estimated between $8 to 16 trillion globally (IPBES, 2020). These figures will continue to rise. The next pandemic is likely to be ‘just around the corner’. This paper has highlighted that the current reliance on international health law, as a reactive measure, is inadequate. Responding to the next zoonotic disease pandemic, requires a significant shift in international agreements to frameworks that synergize environmental and health law. A new generation of human-nature laws, are now required to respond to the multi-facets presented by global challenges, such as epidemics and pandemics.

In this paper, we have provided examples of CHANS-Law which includes greater coordination across international health and environmental agreements. CHANS-Law has the potential to shift the role of international environmental law into one that actively seeks to minimize the likelihood of a future epidemic or pandemic, by addressing the problem at its source. Finally, this paper has mounted the case for a dedicated convention to be developed as a matter of urgency. Considering the 1.7 million potential new virus threats (IPBES, 2020), the next pandemic is likely to be a more virulent and complex pathogen. It is only through pre-emptively protecting the natural environment, that future epidemics and pandemics can be avoided or managed in an integrated manner.

The unpredictable nature of the Anthropocene requires innovative methods for understanding coupled systems and the feedbacks across them. Sophisticated governance systems, capable of anticipating and responding to the novel challenges of global environmental change, are therefore needed. These demand greater integration across traditionally separate sectors and disciplines (Kotchen & Young, 2007).This paper has demonstrated, in the context of epidemics and pandemics, how CHANS-Law, has the capacity to transform the ways in which we approach law. There is the need for greater consideration of hyperconnected global issues through a CHANS-Law lens. It is only by bringing legal disciplines together in recognition of the multilayered complexities of human-nature systems that we might hope to achieve a legal landscape capable of addressing the interconnected challenges of the Anthropocene.

## Footnotes

1 See for example, Adoption of the *Paris Agreement* Arts 4, 7.

2 Australian Government utilized its powers for the first time to manage the pandemic under Section 475 of the *Biosecurity Act 2015* (Cth) (Biosecurity Act (Cth), 2015).
